# The Effect of the Surgical Margins on the Outcome of Patients with Head and Neck Squamous Cell Carcinoma: Single Institution Experience

**DOI:** 10.3969/j.issn.2095-3941.2012.01.005

**Published:** 2012-03

**Authors:** Hany Eldeeb, Craig Macmillan, Christine Elwell, Abdulla Hammod

**Affiliations:** Northamptonshire Centre for Oncology, Northampton, Northamptonshire NN1 5BD, UK

**Keywords:** carcinoma, squamous cell, chemoradiotherapy

## Abstract

**Objective:**

To assess the impact of close or positive surgical margins on the outcome, and to determine whether margin status influence the recurrence rate and the overall survival for patients with head and neck cancers.

**Methods:**

Records from 1996 to 2001 of 413 patients with primary head and neck squamous cell carcinoma (SCC) treated with surgery as the first line treatment were analysed. Of these patients, 82 were eligible for the study. Patients were followed up for 5 years.

**Results:**

Patients with margins between 5-10 mm had 50% recurrence rate (RR), those with surgical margins between 1-5 mm had RR of 59% and those with positive surgical margins had RR of 90% (*P*=0.004). The 5-year survival rates were 54%, 39% and 10%, respectively (*P*=0.002).

**Conclusions:**

Unsatisfactory surgical margin is an independent risk factor for recurrence free survival as well as overall survival regardless of the other tumor and patient characteristics.

## Introduction

Squamous cell carcinoma (SCC) of the head and neck is one of the 10 most frequent malignancies worldwide, with about a quarter of all cases occurring in the developing countries. SCC accounts for more than 90% of all head and neck cancers; it is a malignant tumor of epithelial origin and its behaviour depends on its site of origin. Each anatomic site has its own pattern of spread and prognosis ^[^^1^^]^.

SCC of head and neck accounts for approximately 500,000 new cases worldwide each year ^[^^2^^]^. In Europe, in 1995 there were 72,000 new cases of head and neck SCC and 31,000 deaths, making it the eighth leading cause of cancer death and the seventh for incidence ^[^^3^^]^.

In the UK, about 5,400 new cases were diagnosed in 2007 with the incidence rate significantly higher in Scotland than in other parts of the UK. The incidence is rising; this is thought to be due to HPV related oropharyngeal carcinoma amongst younger people. Although head and neck cancer is more common in men, the sex ratio in the UK dropped sharply from 5:1 fifty years ago to less than 2:1 today ^[^^4^^]^.

The outcome of head and neck cancer depends on many factors, some related to the patient such as the age, performance status, comorbidities and race. Other factors relate to the disease at the time of diagnosis including site, stage, grade and surgical margin status. The treatment decision is guided by these factors and 60% to 65% of patients with early stage disease, stages I and II, can be cured with surgery or radiotherapy, while patients with advanced disease, stages III and IV, need combined treatment modalities with surgery, radiotherapy and chemotherapy. For advanced disease, even with a combined approach, the cure rate is not higher than 30%. The main cause of treatment failure is loco-regional recurrence which occurs in about 60% of patients, followed by distant metastasis in about 30% ^[^^5^^]^.

The impact of the surgical margins on the outcome of head and neck cancer remains equivocal. Some studies showed evidence that these margins were associated with poor outcome in term of disease free survival (DFS) and mortality, but some studies failed to show this impact ^[^^6^^, ^^7^^]^.

## Patients and Methods

This is a retrospective cohort study of head and neck cancer cases diagnosed at Northampton General Hospital, Kettering General Hospital and Milton Keynes Hospital. The patients were treated at Northampton General Hospital at the Oncology Center between 1996 and 2001. The aim of the study is to assess whether the surgical margin status affect the outcome of patients with head and neck cancer at our institute.

The histopathology reports for patients with head and neck SCC that underwent primary surgery for their cancers have been reviewed. Histopathological margins have been categorized into three groups: Group 1: 5-10 mm, Group 2: 1-5 mm, Group 3: positive margin (involved).

The involved margin was defined as: a positive margin in which residual cancer cells were found at the surgical margin by the reporting pathologist.

The time to recurrence, disease free survival (DFS) and overall survival (OS) were assessed. The impact of the surgical margin, lymph node status, tumor size, tumor grading, differentiation and treatment given on the recurrence and overall survival was analysed by univariate and multivariate analysis.

### Inclusion criteria

● Patients who had surgery and were reported by the pathologist to have margins less than 10 mm including the positive margins.

● SCC confirmed by histopathology report.

● Primary site of the tumor was oral cavity, oropharynx, hypopharynx, and larynx.

● Patients treated with surgery only, or surgery followed by radiotherapy and/or chemotherapy.

● No prior treatment of the head and neck cancer.

● Patients treated with radical intent.

● No history or evidence of second malignancy.

● No distant metastasis at the time of diagnosis.

### Exclusion criteria

● Patients reported by the pathologist to have clear margin (more than 1 cm).

● Patients with distal metastasis at the time of diagnosis.

● Patients who received palliative treatment.

● Patients with poor performance status, unsuitable for radical treatment.

● Patients with prior history of malignancy.

The study included patients who were diagnosed between 1^st^ of January 1996 to 31^st^ of December 2001 and had primary surgery. We have reported the surgical margin status as described by the pathologist after resection of the primary tumor. Data on patient follow up was obtained until 1^st^ August 2008. The recurrence and survival was calculated at this point.

The data collected from the patient’s records at Northampton General Hospital included diagnosis date, age at diagnosis, sex, surgery date, surgical margin status, TNM stage, radiotherapy treatment, time to recurrence and date of death.

Data were statistically described in terms of range, mean ± standard deviation (± SD), frequencies (number of cases) and percentages when appropriate. Comparison of recurrence and mortality between the different study groups was done using Chi square (χ^2^) test. Exact test was used in stead when the expected frequency was less than 5. Survival analysis was done for the different outcome measures using Kaplan Maier statistics calculating the mean and median survival time for each group with their 95%CI and the corresponding survival graphs. A probability value (*P* value) less than 0.05 was considered statistically significant. All statistical calculations were done using computer programs Microsoft Excel 2010 (Microsoft Corporation, NY, USA) and SPSS (Statistical Package for the Social Science; SPSS Inc., Chicago, IL, USA) version 15 for Microsoft Windows.

## Results

There were 413 patients with head and Neck SCC registered as new patients at the Oncology Department in Northampton General Hospital through January 1996 to December 2001. More males were diagnosed with head and neck cancers in comparison with females, 60% *vs.* 40%. Of the 413 patients, 337 had radical treatment while 76 patients underwent palliative treatment. Eighty-two patients of the 337 cases met the inclusion criteria, of which 63% were male and 37% were female (59.8% floor of the mouth, 24.4% larynx and 15.8% tonsils). The mean age at diagnosis was 63 years. Patients have been categorized into 3 groups according to the histopathology margins: Group 1: 5-10 mm, Group 2: 1-5 mm, and Group 3: positive margin (involved). Tumor characteristics are shown in **Table 1**, and the surgical margin status is shown in **Table 2**.

**Table 1:** Tumor characteristics.

Characteristics	No. of patients (%)
T status	
T_1_	24 (29.0)
T_2_	19 (23.0)
T_3_	17 (21.0)
T_4_	22 (27.0)
Lymph node status	
N_0_	37(45.0)
N_1_	26 (32.0)
N_2_	19 (23.0)
Histopathology status	
Well differentiation	24 (29.0)
Moderate differentiation	40 (49.0)
Poor differentiation	18 (22.0)

**Table 2:** The surgical margin status.

Surgical margin status	No. of patients (%)
5-10 mm	30 (36.6)
1-5 mm	32 (39.0)
Positive margin	20 (24.4)

### Recurrence

**Figure 1:**
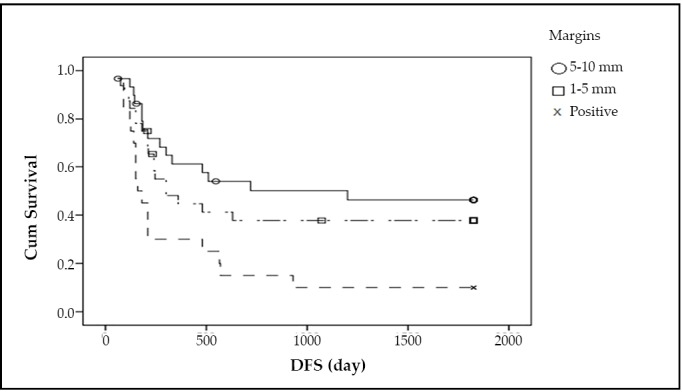
The recurrence rate.

**Figure 2:**
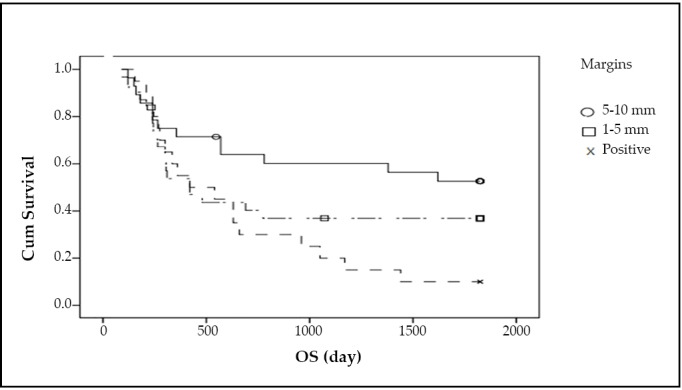
The median survival.

The recurrence rate for Group 1, 2 and 3 was 50%, 59.4%, and 90%, respectively. The median DFS in these groups was 30, 25, and 13 months, respectively. The local recurrence rate was 27%, 36%, and 53%, respectively. Regional recurrence rate in the three groups was 19%, 18%, and 26%, respectively. Distant metastasis occurred in 4%, 5% and 11% cases in the three groups, respectively. The recurrence rate in the positive margin group was significantly higher than in the other two groups (*P*=0.004) (**Figure 1**). The median survival for Group 1, 2 and 3 was 36, 26, and 20 months, respectively, and the mortality rates in these groups were 43%, 59%, and 90%, respectively (*P*=0.002) (**Figure 2**).

The impact of tumour size on DFS and OS is shown in **Table 3**, the impact of lymph nodes involvement (N stage) on DFS and OS is shown in **Table 4**, and the impact of tumor grading on DFS and OS is shown in **Table 5**.

**Table 3:** Impact of tumor size on DFS and OS.

T status	No. of patients	Recurrence (%)	Mortality (%)
T_1_	24	13 (54.0)	11 (46.0)
T_2_	19	7 (37.0)	7 (37.0)
T_3_	17	14 (82.0)	14 (82.0)
T_4_	22	18 (81.0)	18 (81.0)

DFS, *P*=0.002; OS, *P*=0.002

**Table 4:** Impact of Lymph Nodes status on DFS and OS.

N status	No. of patients	Recurrence (%)	Mortality (%)
N_0_	37	18 (48.0)	16 (44.0)
N_1_	26	18 (69.0)	18 (69.0)
N_2_	19	16 (84.0)	16 (84.0)

DFS, *P*=0.005; OS, *P=*0.001

**Table 5:** Impact of tumour grading on DFS and OS.

Differentiation	No. of patients	Recurrence (%)	Mortality
Well	24	14 (58.0)	12 (50.0)
Moderate	40	23 (57.0)	23 (57.0)
Poor	18	15 (83.0)	15 (83.0)

DFS, *P*=0.08; OS, *P=*0.003

Fifteen patients underwent surgical treatment only, while 67 patients underwent surgery followed by adjuvant radiotherapy. The recurrence rate was 40% in the first group and 68.7% in the second group. As one of the indications for postoperative radiotherapy, positive surgical margins may be the reason why more recurrences occurred in those who received adjuvant radiotherapy. Also, most patients treated with surgery alone had early stage disease (*P*=0.04).

The overall survival rate in the group who underwent surgical treatment alone was 54%, while those who underwent surgery followed by radiotherapy showed a survival rate of 33% (*P*=0.10). Multivariate analysis (**Table 6**) showed that the surgical margin persisted as a survival predictor regardless of the tumor characteristics at the time of diagnosis (*P*=0.02). Tumor size (*P*=0.02) and lymph node status (*P*=0.03) were also prognostic factors. Interestingly the multivariate analysis showed that the tumor grading was the most influential prognostic factor affecting the DFS and OS (*P*=0.001).

**Table 6:** Multivariate analysis of factors affecting DFS and OS.

Factors	*P* (DFS)	*P* (OS)
Margin	0.07	0.02
Tumor size, T	0.03	0.02
Lymph nodes, N	0.06	0.03
Performance status	0.4	0.4
Site	0.5	0.2
Grading	0.001	0.001

## Discussion

Head and neck cancers are the sixth most common cancer in the world and SCC represents the most common pathological type ^[^^8^^]^.

In our study, we have included only patients who underwent surgery as the first treatment modality and excluded those who had neoadjuvant chemotherapy or radiotherapy or both. Patients who had a surgical margin more than 1 cm were excluded as nearly all of the relevant studies reported margin status between 3-5 mm, with 1 cm being accepted as a generous margin ^[^^9^^]^.

The most generous margin recommended is 8-10 mm, which has been attributed to what is called the “formalin-shrinkage” effect which can be at least 30% ^[^^10^^]^. It has been claimed that surgeons need to ensure 8-10 nm *in situ* surgical margin to get 5 mm pathological clearance ^[^^11^^]^.

The UK guidelines consider margins of 5 mm and more as clear, 1-5 mm as close and less than 1 mm as involved margins ^[^^12^^]^. In our study the recurrence rate was 50% in the group of margins between 10 mm to 5 mm, 59.4% in those with margins between 5 mm to 1 mm. In the group with positive margins, the recurrence rate was 90% (*P*=0.004). The mortality rates in these groups were 43%, 59%, and 90%, respectively (*P*=0.002). This result was compatible with the result of Nason et al. 2009 ^[^^13^^]^, which showed that the survival improved with each additional millimetre of clear surgical margin, and patients with margins of 5 mm or more had a 5-year survival rate of 73% when compared to those with margins of 3 to 4 mm (69.0%), 2 mm or less (62.0%), and positive margins (39.0%), *P*=0.000. Another study showed that positive surgical margins decreased the 5-year survival (*P*=0.02), and were significantly associated with time to tumor recurrence (*P*=0.001) ^[^^14^^]^.

The major site of lesions in our study was the floor of mouth, which indicated primary surgery is indicated and where obtaining a margin more than 1 cm was difficult. Primary surgery for advanced laryngeal cancer was the standard during the study period. Tonsillar cancers were mainly treated with either neo-adjuvant chemotherapy followed by either surgery or radical chemoradiotherapy leaving only a small proportion of them treated with primary surgery eligible in our study.

No laser treatment was used in any patients in this study. There was no documentation on any patient regarding a re-excision. Cancer of the floor of mouth accounted for most of the cases, resulting in higher recurrence rates and negative effect on OS. In a study of 115 T_1_-T_2_ oral squamous cell carcinoma cases, Jerjes et al. ^[^^15^^]^ reported a recurrence rate of 43%.

The impact of the disease characteristics at the time of diagnosis is well documented in most of studies. TNM staging and histopathology grading strongly correlate to the outcome.

In our study, the recurrence rates for T_3_ and T_4_ tumors were 82% and 81%, respectively, while those of T_1_ and T_2_ were 54% and 37%, respectively (*P*=0.002). The mortality rates in the same population were 46%, 37%, 82% and 81% for T_1_, T_2_, T_3_ and T_4_, respectively (*P*=0.002). A study showed the same result in regard to impact of tumor size on the outcome. The earlier was the tumor stage, the better was the prognosis ^[^^16^^]^. The local control rate in another study was 3 times lower for advanced disease (33% for T_3_) in comparison with 93% for early disease of T_1_^[^^17^^]^. The same picture is true for lymph node involvement. The more lymph nodes were involved, the worse the prognosis was. We found that the recurrence rates were 48%, 69% and 84% for N_0_, N_1_ and N_2_, respectively (*P*=0.005). Mortality rates were 44%, 69%, and 84% for N_0_, N_1_, and N_2_, respectively (*P*=0.001). Greenberg concluded the same results and showed that there were statistically significant differences in term of DFS and OS in association with lymph node involvement, and the regional lymph node metastasis was the most reliable predictor of treatment outcome ^[^^18^^]^.

Histological grading was found to be a significant predictor for treatment failure and recurrence^[^^19^^]^. In another study it was related to nodal disease at the time of diagnosis and influenced the outcome significantly ^[^^20^^]^, but this was not the case in our series. In our study, almost 50% of the patients presented with moderately differentiated carcinoma and the recurrence rate in this group was 57.0%, while in well differentiated carcinoma group, the recurrence rate was 58%. The poorly differentiated cases showed the worse prognosis with the highest recurrence rate of 83.0% (*P*=0.08). Mortality rates in these groups were 50.0%, 57.0%, and 83.0% respectively (*P*=0.03).

The type of treatment given has not influenced the outcome significantly, as adjuvant radiotherapy was offered for selected patients with either close (<2 mm) or positive margins or advanced disease. No adjuvant chemoradiotherapy was given during 1995 and 2001. The adjuvant radiotherapy dose given was 60 Gy/30 fractions/6 weeks. The criteria for using adjuvant radiotherapy were positive tumor margin, T_4_ lesion and 2 or more involved lymph nodes.

Although there is growing evidence and indications for use of adjuvant chemoradiotherapy ^[^^21^^]^, a wide range of inconsistency still exists in clinical practice ^[^^22^^]^.

The updated European treatment recommendations for management of SCC of the head and neck have recommended the use of adjuvant chemoradiotherapy in patients with positive margins or extra capsular spread. However, adjuvant radiotherapy was only recommended for stage III, IV, perineural involvement or vascular tumor embolism ^[^^23^^]^.

Patients with clear margins or early stage disease underwent only surgical treatment and none of them had positive margins. This prevents us from making any meaningful judgment on the impact of radiotherapy and chemotherapy on patients’ outcome.

## Conclusion

Despite the progress in treatment modalities, head and neck cancers still carry a poor prognosis in term of DFS and OS. The outcome of head and neck cancers depends on many factors like the TNM staging, site of the tumor, tumor differentiation, and performance status. This study concluded that there is significant impact of positive surgical margins on the outcome in univariate and multivariate analysis and that adjuvant radiotherapy/chemotherapy cannot fully compensate for the close and positive margins. We could not draw any conclusions regarding the outcome of patients with margin >1 cm as they were excluded from this study.
